# Genome-wide miRNA profiling reinforces the importance of miR-9 in human papillomavirus associated oral and oropharyngeal head and neck cancer

**DOI:** 10.1038/s41598-019-38797-z

**Published:** 2019-02-19

**Authors:** Ksenija Božinović, Ivan Sabol, Emil Dediol, Nina Milutin Gašperov, Spomenka Manojlović, Zuzana Vojtechova, Ruth Tachezy, Magdalena Grce

**Affiliations:** 1 Division of Molecular Medicine, Ruđer Bošković Institute, Zagreb, Croatia; 2 Clinical hospital Dubrava, Department of Maxillofacial Surgery, Zagreb, Croatia; 3 Department of Genetics and Microbiology, Faculty of Science, Charles University, BIOCEV, Vestec, Czech Republic

## Abstract

Head and neck cancer is the sixth most common malignancy worldwide, predominantly developing from squamous cell epithelia (HNSCC). The main HNSCC risk factors are tobacco, excessive alcohol use, and the presence of human papillomavirus (HPV). HPV positive (+) cancers are etiologically different from other HNSCC and often show better prognosis. The current knowledge regarding HNSCC miRNA profiles is still incomplete especially in the context of HPV+ cancer. Thus, we analyzed 61 freshly collected primary oral (OSCC) and oropharyngeal (OPSCC) SCC samples. HPV DNA and RNA was found in 21% cases. The Illumina whole-genome small-RNA profiling by next-generation sequencing was done on 22 samples and revealed 7 specific miRNAs to HPV+ OSCC, 77 to HPV+ OPSCC, and additional 3 shared with both; 51 miRNAs were specific to HPV− OPSCC, 62 to HPV− OSCC, and 31 shared with both. The results for 9 miRNAs (miR-9, -21, -29a, -100, -106b, -143 and -145) were assessed by reverse transcription-quantitative polymerase chain reaction on the whole study population. The data was additionally confirmed by reanalyzing publicly available miRNA sequencing Cancer Genome Atlas consortium (TCGA) HNSCC data. Cell signaling pathway analysis revealed differences between HPV+ and HPV− HNSCC. Our findings compared with literature data revealed extensive heterogeneity of miRNA deregulation with only several miRNAs consistently affected, and miR-9 being the most likely HPV related miRNA.

## Introduction

Head and neck cancer (HNC) is the sixth most common malignancy worldwide, predominantly arising within the mucosal linings of the upper aerodigestive tract^1^. Most HNC develop from squamous cell epithelia, which accounts for 95% of head and neck carcinoma (HNSCC)^2,3^. HNSCC often gets diagnosed in a late phase, when it is difficult to treat, with 5-year survival of only 40–50%^4,5^. HNSCC are further characterized according to their primary site of origin, with most common sites being oral cavity, oropharynx, pharynx, larynx, and sinonasal tract^6^. Globally, HNSCC accounts for approximately 550,000 cases annually^7^, while in Croatia, 896 new cases were estimated in 2015^8^.

The main risk factors for HNSCC development are smoking and excessive alcohol use. Furthermore, the role of human papillomavirus (HPV) has emerged in recent years, particularly in oropharyngeal tumors^9,10^. In western countries, tobacco and alcohol induced HNSCC is declining, while HPV-driven HNSCC, especially oropharyngeal, is increasing in younger individuals^9,10^. HPV type 16 has been found in the majority of HPV associated HNSCC, and it is capable of transforming infected cells into cancerous by expressing oncoproteins E6 and E7, which bind, among others, to two important tumor suppressor proteins, p53 and pRB, respectively^10^.

Based on the HPV presence, HNSCC is broadly divided in two groups: HPV positive (+) with better prognosis and HPV negative (−) tumors with worse prognosis^10^. Even though these two groups are etiologically different, the treatment remains the same^7^. However, there are indications that the treatment could be optimized for each groups of patients. Therefore, it is crucial to find more sensitive and specific biomarkers, which could enable development of better diagnostic, prognostic and therapeutic approaches for HNSCC. The HPV positive oropharyngeal cancer in particular was found to be so different from other HNSCC subtypes that the new TNM classifications^11^ and the specific staging guidelines^12^ were made specifically for this subset of tumors. In 2015, The Cancer Genome Atlas (TCGA) consortium published a comprehensive molecular catalogue on HNSCC^13^. Frequent mutations of novel druggable oncogenes were not demonstrated, but the difference between the HPV associated and non-viral groups was confirmed. The TCGA study revealed that HNSCC lacked predominant gain-of-function mutations in oncogenes, whereas an essential role of epigenetics in oncogenesis has become apparent. The study of Masuda *et al*.^14^ emphasizes that HNSCC seems to be an epigenetic disease, rather than genetic. Studies on the epigenetic changes in HNSCC such as miRNA profiling are promising to find specific biomarkers for both groups of tumor patients^15^.

Small non-coding RNAs, such as miRNA (miR) are highly conserved and about 22 nucleotides long, with important role in a variety of processes, including development, cell proliferation, and differentiation^16^. Previous reviews^17–21^ have already noticed a discrepancy of miRNA findings across studies focusing on HNC, and there is no consensus on specific miRs that are associated to HPV+ HNSCC. Therefore, more thorough studies on a homogeneous and well described study population, with distinguishing tumor site of origin and etiology are necessary to get a clearer picture.

In this study, we collected and analyzed fresh tissue of primary oral and oropharyngeal HNSCC patients, determined the HPV status and performed whole-genome miRNA profiling to identify differences in miRNA expression levels. We also performed a literature review on similar medium and high throughput miRNA studies on HNSCC populations to evaluate our findings in this context. Furthermore, we have reanalyzed the publicly available TCGA miRNA sequencing data in the same context to further validate our findings.

## Material and Methods

### Patient material

HNSCC patients with primary oral (O) and oropharyngeal (OP) tumors treated at the Clinic of Maxillofacial Surgery of the Clinical Hospital Dubrava between 2015 and 2017 were enrolled in the study. The study was approved by the Bioethical Board of the Ruđer Bošković Institute (BEP-3748/2-2014) and the Ethical Board of the Clinical Hospital Dubrava (EP- KBD-10.06.2014). The experiments were performed in accordance with relevant guidelines and regulations. Informed consent to participate in the study was obtained for 65 cases. Four cases were excluded because 2 tumors were not primary and 2 were not squamous cell carcinoma. Thus, the total of 61 primary cancer samples have been included in this study. The majority (75%) of tumors were of oral origin (tongue, floor of mouth, buccal mucosa, gingiva and retromolar region), while a quarter were of oropharyngeal origin (base of tongue, tonsil and posterior pharyngeal wall).

All patients underwent surgery as primary treatment and 2 pieces of tumor tissue were stored in 700 μL of G2 lysis buffer (Qiagen) or RNA later (Ambion) solutions for the isolation of DNA and RNA, respectively. Samples were stored at +4 °C and delivered to the laboratory at the end of the day where they were stored at −20 °C for up to 2 weeks before DNA and RNA isolation.

To supplement the samples, the analysis included 3 additional HPV+ (DNA and RNA) tonsil carcinoma samples and 3 additional HPV− normal tonsillar tissue, collected at the Motol University hospital (Prague, Czech Republic) and previously analyzed by TaqMan miRNA Cards A and B^22^.

### Nucleic acid isolation

For nucleic acid extraction, smaller (~20 mg) pieces of tumor tissue were used. The DNA was isolated with the EZ1 Biorobot using EZ1 DNA tissue kit (Qiagen) following the manufacturer’s protocol. The total RNA was isolated using miRNeasy mini kit (Qiagen) also following the manufacturer’s protocol. Both DNA and RNA quality and quantity were analyzed on nanospectrofotometer (Implen). Furthermore, RNA quality was assessed by Bioanalyser RNA6000 Nano kit (Agilent) to determine the RIN number.

### HPV analysis

The presence of HPV DNA was assessed using three types of consensus and one type specific PCR primer pair as described previously^23^. Briefly, 50 ng of sample DNA was amplified by PGMY, GP and/or SPF10 primers and with primers specific for HPV16. To determine sample adequacy human beta-globin specific PCR was performed. Samples with any discrepancy (n = 14) in HPV testing were further analyzed using INNO-LiPA HPV Genotyping Extra (Fujirebio) according to the manufacturer’s protocol.

HPV16 E6 mRNA analysis was performed on HPV DNA positive samples. Briefly, one μg of RNA was reverse transcribed using QuantiTect Reverse Transcription kit (Qiagen) according to the manufacturer’s protocol. The presence of full length or most abundant splice variant of the HPV16 E6 open reading frame (E6*I) was detected by PCR^24^ and the amplicons (~260 and ~86 bp, respectively) were visualized on 3%-agarose gel electrophoresis. CaSki cell line cDNA was used as positive control, while the negative control contained all PCR reagents without cDNA. The suitability of cDNA for amplification was confirmed by beta-actin PCR^25^. In this study, samples positive for both HPV DNA, and E6 mRNA were considered HPV positive.

To try separating relevant HPV infections from those where other factors might confound HPV activity, patients were also classified into risk groups according to Ang *et al*.^26^. Briefly, low risk group is defined as HPV positive tumors from non-smoking patients or from smoking patients with lower nodal stage. High risk group consists of HPV negative smokers or tumors with high T classification in nonsmokers, while the intermediate risk group consisted of smoking patients with HPV positive N2b+ tumors or non-smoking HPV negative tumors with T classification less than 4.

### miRNA next generation sequencing analysis

A subset of samples (19 cancer samples and 3 controls) was selected for high-throughput miRNA analysis by next generation sequencing (NGS). Samples with poor RIN (<7) scores were excluded. Thus, the following samples were selected for NGS library preparation: 6 HPV+ (DNA and RNA) and 4 HPV− oropharyngeal cancer samples (OP+ and OP−, respectively); 3 HPV+ (DNA and RNA) and 6 HPV− oral cancer samples (O+ and O−, respectively); and 3 healthy tonsil tissue samples (controls). Out of 6 selected OP+ samples, two were classified as intermediate risk group according to Ang *et al*.^26^ Twenty-two NGS libraries were constructed with TrueSeq Small RNA Library prep kit (Illumina) according to the manufacturer’s protocol. For multiplexing and library pooling, index pools A (1–12) and B (13–22) were used. Bioanalyser (Agilent) was used for quality control of indicated steps as recommended by the manufacturer. Library sequencing was done on NextSeq 500 sequencer (Illumina) using NextSeq 500 Mid output kit (Illumina).

Raw sequences were trimmed of adapter sequences using FastQ toolkit Basespace App (Illumina) by selecting TrueSeq Small RNA adapter sequences from the relevant app menu. Sequencing data was analyzed using Small RNA Basespace App v1.0.1 (Illumina) to determine significantly different miRNA expression between groups. The automated pipeline uses Bowtie to align reads against reference databases to determine counts, which are then assessed for differential expression using DESeq2. Within the pipeline, miRNA sequences with mean normalized counts across all samples ≤10 are filtered out before statistical analysis. Further analyses were performed by importing Small RNA Basespace App count data into R and independently analyzed by the DESeq2 package.

### Technical validation

The NGS results were validated by real-time quantitative Reverse Transcription PCR (qRT-PCR) on the same samples tested by NGS. For technical validation of the NGS experiment, we have selected 9 miRNAs that were found to be differentially expressed by NGS (miR-9-5p, -21-3p, -27a-5p, -31-5p, -34a-5p, -100-5p, -143-3p, -145-5p, 218-5p). Assays were designed to cover both over and under expressed miRNA. Priority was given to miRs found in HPV positive samples but without being found as significant in HPV negative samples. In addition, miRNAs -21-3p, -31-5p, -100-5p were chosen, since they were often reported in many different cancer types and could represent positive control targets. The TaqMan Advanced miRNA synthesis kit (Applied Biosystems) was used to convert isolated RNA to cDNA following the manufacturers protocol. Following conversion, 5 μl of diluted cDNA was analyzed by reverse transcription-quantitative polymerase chain reaction (RT-qPCR) using TaqMan Advanced miRNA single tube assays (Applied Biosystems). The three normal tonsillar samples were pooled in equal concentration before cDNA synthesis to be used as normal reference. Assays for miR-16-5p and -191-5p were evaluated as internal reference control (manufacturer’s recommendation) as well as miR-181a-5p that showed very low intra-sample variation in the NGS experiment. Calculations were performed using each of the 3 referent miRs individually (data not shown) and as average of all 3 values. As the results were similar, the final analysis was performed with the average value of all 3 reference miRs. The fold changes were calculated using the standard 2^−ddCt^ method^27^. Briefly, dCt values were obtained by normalizing to the referent control sample, i.e. obtained by subtracting mean replicate Ct values of the combined referent sample from the mean replicate Ct value of each sample for each miR tested. Subsequently, dCt values for each miR were normalized to referent miRs in each sample to obtain ddCt value. The fold change was calculated by 2^−ddCt^ formula. The statistical difference was tested by t-test on dCt values of each miR compared to dCt values of the referent miR within each subgroup of samples.

### Clinical validation

For further validation of potentially relevant miRs, clinical samples not tested by NGS (independent set of 46 tumors and same controls used for technical validation) were tested with qRT-PCR individual assays in the same way as for technical validation. For clinically relevant validation, priority was given to miRNAs with at least 100 normalized mean count in OP+ subset since low count (expression) might lead to inconsistent results on routine samples. Even though NGS analysis indicated a very limited number of miRNAs exclusively associated with HPV, the following miRNAs were selected for analysis: miR-9-5p, -21-3p, -29a-3p, -100-5p, -106b-5p, -143-3p, and -145-5p. The miRNAs miR-9-5p, -106b-5p and -29a-3p were selected as they were deregulated in our OP+ subset and not found significant in HPV negative samples. As for technical validation, miRs -21-3p and -100-5p were chosen because of their relevance in different cancer types. Specifically miRs -143-3p and -145-5p were selected as they were most commonly found by other studies to be downregulated in HNSCC cases even though they were not found to be significant in our HPV positive samples. In both cases, the purpose was to assess the utility of selected miRs as potential biomarkers. Internal reference controls and combined sample pool of healthy tonsil samples was used as referent sample for fold change calculations; as done for the technical validation. Since the initial NGS set was selected with overrepresentation of HPV positive and oropharyngeal samples, the independent set was underrepresented in those samples and consisted of 35 O−, 6 OP−, 3 O+ and only 2 OP+ samples. To increase robustness, analysis was done on the independent set (n = 46) or the total set (n = 61) of clinical cancer samples.

### Independent validation

To assess the validity of the results in a completely unrelated set of patients, we accessed publicly available miRNA sequencing data from TCGA data portal for oral and oropharyngeal cancer samples. Detailed clinical data and HPV status for cases with available miRNA sequencing data was obtained from the TCGA data portal as well as the TCGA consortium HNSCC focused publication^13^. We were able to match miRNA sequencing data and relevant information for 72 cancer samples (Supplementary Dataset SD1). There were 40 samples from oral cancer (12 HPV RNA positive) and 32 oropharyngeal cancer (21 HPV RNA positive) patients. We were also able to find miRNA sequencing results for matched normal solid tissue from two oropharyngeal cancer and two oral cancer patients. However, we chose to include only oropharyngeal tissue normal controls to make the control groups comparable to our sequencing experiment, where we have also used oropharyngeal normal samples as control. Briefly, raw counts of all miRNA sequences were tabulated (including isomiR sequences) and imported to R alongside annotation data (Supplementary Dataset SD1) for the analysis with DESeq2 package using identical R pipeline as for our samples. As before, miRNA sequences with 10 or less normalized reads across all samples were removed. Samples were also additionally classified according to Ang *et al*.^26^ risk factors from available clinical data, which included smoking and pack/year data for the majority of cases.

### miRNA classifier

In attempt to create a miRNA classifier from the NGS data, we used multinomial sparse group lasso method as implemented within msgl R package^28,29^. Normalized counts of our and TCGA data were imported to R as “reads per million miRNA mapped”. Our sequencing data was used either as a training set for TCGA data classification, or as test set after training the classifier on TCGA data. Another set of classifier models were created where TCGA dataset was split in half with the first half used for training and the second for testing. Classification was performed for several variables: sample group (OP+, OP−, O+ and O−), HPV RNA presence (HPV+, HPV−) and risk group (high, intermediate, low) according to Ang *et al*.^26^.

### Statistical analysis

Data management and basic analysis was done in Microsoft Excel, while statistical testing was done in Medcalc (v 11.4.2). R studio (v 1.1.383) was used to interface with R (v 3.4.2.) and perform miRNA differential expression using DEseq2 (1.18.1)^30^ or msgl classifier training and testing.

### Literature review

So far, overlap of published results on miRNA deregulation in HNSCC, when only the validated or the most relevant miRs from each manuscript are considered, is relatively low^17,18,20,21^. Thus, to help us determine the relevance of particular miRNAs we decided to reexamine complete data from medium- and high-throughput miRNA HNSCC studies. To this end, we searched the NCBI PubMed database for such studies using combinations of terms HNSCC, HNC, head and neck cancer, OPSCC, oropharyngeal, miRNA, microRNA, small RNA, microarray, NGS, and next generation sequencing. Studies were accessed and wherever possible raw or supplemental data including all significantly deregulated miRNAs were extracted and tabulated. Where data was not available in the manuscript or Supplement material, we sent queries to the corresponding authors but rarely got answers. Where the information regarding -5′ or -3′ strand was not given, we considered the miRNA to belong to the more abundant form according to miRbase. If a miRNA was marked with the asterisk (*), we considered it to correspond to less abundant form according to miRbase. In addition, included articles as well as published reviews referring to the topic were searched for references to primary publications. However, reviews themselves were not included unless summarizing primarily low-throughput studies. Thus, care has been taken to include each primary study only once within the final Table (Supplementary Dataset SD2). The exception was in case when a particular study presented the differential expression results of several comparisons in which case significantly deregulated miRNAs from each comparison are listed as a separate column. In addition, studies examining miRNA deregulation in cervical cancer but included in the HNSCC focused reviews were also included to allow cross tissue examination of HPV infection related miRNAs. The prepared table (Supplementary Dataset SD2) served to both demonstrate heterogeneity of previous studies (Supplementary literature), to inform decisions for the current study, and to put the results in context.

## Results

### Patients

A total of 61 primary oral and oropharyngeal cancer samples were included in the study. Cases were grouped according to the HPV presence and patient characteristics are summarized in Table 1. The majority of patients were male (77.0%), had history of smoking (65.6%) and were treated for advanced stage disease (stage III or worse; 73.8%). On average, the patients were 62.7 years old (age range 31–85) with a median of 62 years. HPV DNA was found in 14 of 61 samples (22.9%). Only 2 samples contained HPV 18 DNA, while the remaining 12 contained HPV16 DNA. HPV 16 RNA was found in 11 of 12 (91.7%) HPV 16 DNA positive samples. Approximately 21.3% cancer samples contained transcriptionally active HPV with the percentage being the highest in OPSCC samples (31.3%) and in particular in tonsillar cancer (57.1%). HPV+ patients were slightly younger than HPV− patients (average of 59.8 vs 63.4 years) but the difference was not statistically significant, and neither were the median ages (62.5 and 62 years). The majority were classified as high risk group (63.0%), while only 10 (16.4%) were classified as low risk according to the criteria by Ang *et al*.^26^; only 3 HPV positive samples were classified as intermediate risk.

**Table 1:** Description of the study population.

Characteristics		HPV positive (DNA and RNA) (n = 13)	HPV negative (n = 48)	Total (n = 61)
		N (%)	N (%)	N (%)
Gender	Male	11 (84.6%)	36 (75%)	47 (77%)
	Female	2 (15.4%)	12 (25%)	14 (23%)
Age	Median	64	62	62
	0–45	2 (15.4%)	0 (0%)	2 (3.3%)
	45–64	5 (38.5%)	27 (56.3%)	32 (52.5%)
	65+	6 (46.2%)	21 (43.8%)	27 (44.3%)
Life style factors	NSND	5 (38.5%)	16 (33.3%)	21 (34.4%)
	S	1 (7.7%)	3 (6.3%)	4 (6.6%)
	SD	7 (53.8%)	29 (60.4%)	36 (59%)
Tumor Location	Oropharynx	5 (31.3%)	11 (68.8%)	16 (26.7%)
	Tonsil	4 (57.1%)	3 (42.9%)	7 (11.7%)
	Base of tongue	1 (14.3%)	6 (85.7%)	7 (11.7%)
	Oropharyngeal wall	(0%)	2 (100%)	2 (3.3%)
	Oral	8 (17.8%)	37 (82.2%)	45 (75%)
	Gingiva	2 (14.3%)	12 (85.7%)	14 (23.3%)
	Floor of mouth	1 (8.3%)	11 (91.7%)	12 (20%)
	Tongue	4 (40%)	6 (60%)	10 (16.7%)
	Retromolar	1 (14.3%)	6 (85.7%)	7 (11.7%)
	Buccal mucosa	(0%)	2 (100%)	2 (3.3%)
Clinical T stage	1	2 (15.4%)	4 (8.3%)	6 (9.8%)
	2	2 (15.4%)	16 (33.3%)	18 (29.5%)
	3	3 (23.1%)	12 (25%)	15 (24.6%)
	4	6 (46.2%)	16 (33.3%)	22 (36.1%)
Clinical N stage	0	5 (38.5%)	22 (45.8%)	27 (44.3%)
	1	3 (23.1%)	15 (31.3%)	18 (29.5%)
	2	4 (30.8%)	6 (12.5%)	10 (16.4%)
	3	1 (7.7%)	5 (10.4%)	6 (9.8%)
Overall stage	Early stage	2 (15.4%)	14 (29.2%)	16 (26.2%)
	I	(0%)	2 (4.2%)	2 (3.3%)
	II	2 (15.4%)	12 (25%)	14 (23%)
	Late stage	11 (84.6%)	34 (70.8%)	45 (73.8%)
	III	2 (15.4%)	12 (25%)	14 (23%)
	IVa	8 (61.5%)	17 (35.4%)	25 (41%)
	IVb	1 (7.7%)	5 (10.4%)	6 (9.8%)
Tumor grade	1	4 (30.8%)	19 (39.6%)	23 (37.7%)
	2	5 (38.5%)	20 (41.7%)	25 (41%)
	3	3 (23.1%)	4 (8.3%)	7 (11.5%)
	Unknown	1 (7.7%)	4 (8.3%)	5 (8.2%)
Risk group	High	0 (0%)	38 (79.2%)	38 (62.3%)
	Intermediate	3 (23.1%)	10 (20.8%)	13 (21.3%)
	Low	10 (76.9%)	0 (0%)	10 (16.4%)

### Illumina TrueSeq Small RNA sequencing

To analyze miRNA differences in expression profiles, a total of 22 NGS sequencing libraries were made from: 6 HPV+ and 4 HPV− oropharyngeal cancer samples (OP+ and OP−); 3 HPV+ and 6 HPV− oral cancer samples (O+ and O−); and 3 normal tonsil tissue samples. Complete sample annotation table is provided as Supplementary Table S1. The sequencing run generated approximately 110 million reads that passed QC filter with 96.4% bases having Q30 or greater score. All four sets of cancer tissues were analyzed for differential expression in comparison to control samples (R; DESeq2). Sample distance and principal component analysis (PCA) plots indicated that 2 samples behaved as outliers and were thus excluded from subsequent analysis (indexes 9 and 16, corresponding to one OP− and one O− sample).

Final analysis revealed differential regulation of 501 different unique miRNA sequences (adjusted p value < 0.05) which were significantly deregulated in 1154 comparisons across all sample group comparisons (Supplementary Dataset SD3). However, out of 501 unique sequences only 101 fully corresponded to mature miRNA sequences, while the rest were isomiRs with either sequence mismatches or with slightly different size.

**Figure 1:**
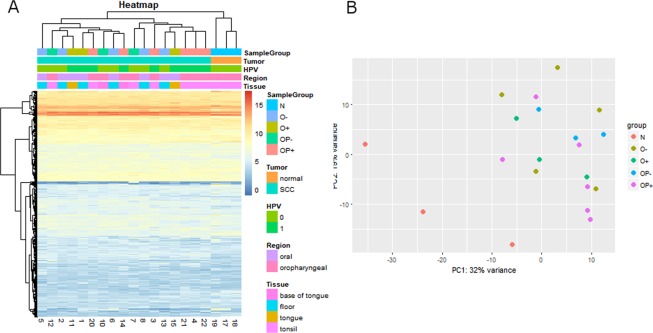
Clustering of the study population on heatmap (**A**) and PCA (**B**). All miRNAs with normalized mean count >10 are included and represented as individual horizontal lines on heatmap. Only separation of normal and tumor groups can be clearly seen. HPV = human papillomavirus, HPV status is determined based on DNA and RNA positivity, HNSCC = head and neck squamous cell carcinoma, N = control, healthy tonsil samples, O− = HPV negative oral SCC samples, O+ = HPV positive oral SCC samples, OP− = HPV negative oropharyngeal SCC samples, and OP+ = HPV positive oropharyngeal SCC samples.

**Figure 2:**
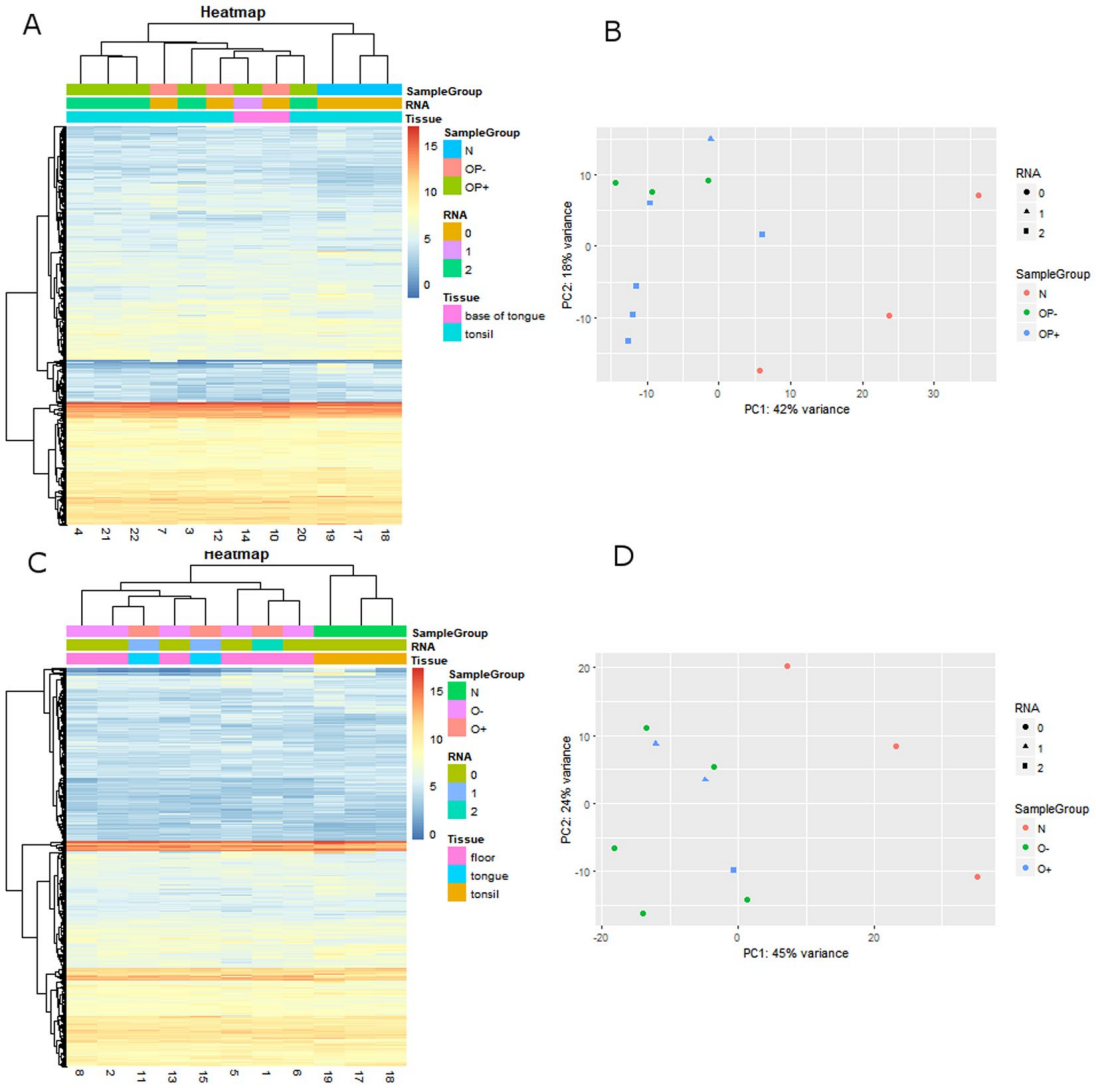
Clustering of the samples separated into oropharyngeal (OP) and oral (O) subgroups (panels AB, and CD) on heatmap (**A,C**) and PCA (**B,D**) plots. All miRNAs with normalized mean count >10 are included and represented as individual horizontal lines on heatmaps. Better but suboptimal separation of oropharyngeal cancer samples based on HPV can be seen. No HPV E6 mRNA presence is indicated by RNA 0 (circle), unspliced mRNA form by 1 (triangle) and fully spliced E6*I form by 2 (square). HPV = human papillomavirus, HPV status is determined based on DNA and RNA positivity, SCC = squamous cell carcinoma, N = control, healthy tonsil samples, O− = HPV negative oral SCC samples, O+ = HPV positive oral SCC samples, OP− = HPV negative oropharyngeal SCC samples, and OP+ = HPV positive oropharyngeal SCC samples.

Based on the final results and on all miRNA sequences, samples did not form distinguishing clusters on heatmap or PCA plots (Fig. 1) except for clear separation of control samples. By separating the samples in oropharyngeal samples and oral sample subgroups, relevant subgroups become better distinguished on the same heatmap and PCA plots (Fig. 2). Classifying samples based on risk levels also failed to separate samples on PCA plots (Supplementary Fig. S1).

### miRNA specific to sample subgroups

**Figure 3:**
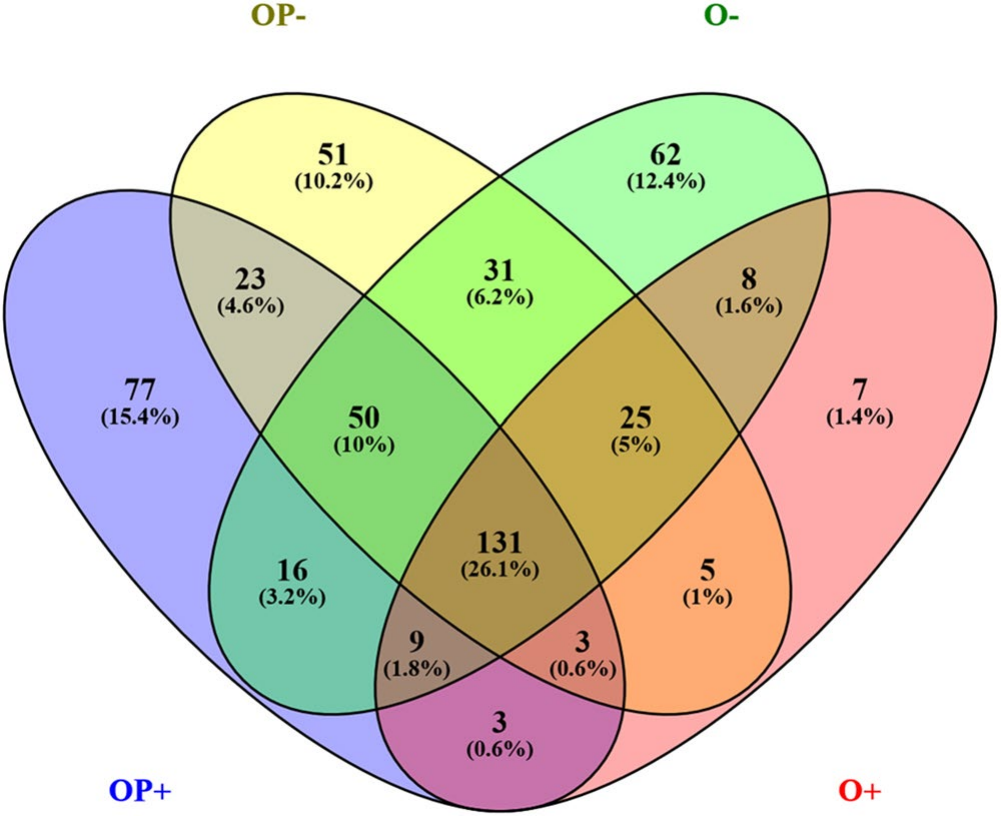
Venn diagram of significantly differentially deregulated miRNA sequences across sample groups. HPV = human papillomavirus (based on DNA and RNA positivity), SCC = squamous cell carcinoma, OP+ = HPV positive oropharyngeal SCC samples, OP− = HPV negative oropharyngeal SCC samples, O+ = HPV positive oral SCC samples, and O− = HPV negative oral SCC samples.

The lists of significantly deregulated miRs in each sample subgroup were compared with Venny 2.1 tool^31^ (Fig. 3). Of all significantly deregulated miRs, 77 were specific to HPV+ oropharyngeal cancer sample group with an additional 3 shared with HPV+ oral cancer group (Supplementary Dataset S4), while only 7 miRNAs were specific to HPV+ oral cancer group. In addition, 51 miRs were specific to HPV− oropharyngeal cancer sample group, 62 to HPV− oral cancer sample group, and 31 shared with both. However, of the combined 80 significantly deregulated miRs only 16 corresponded to mature miRNAs (miR-9-5p, 25-5p, -29a-3p, -29b-3p, -34a-5p, -93-5p, 106b-5p, -133a-5p, -133a-3p, -139-5p, -140-5p, -147b, -208b-3p, 210-5p, 328-3p, -1307-3p), while the rest corresponded to different isomiRs of 42 individual miRNAs (Supplementary Dataset S4).

### Technical validation

Technical assay validation was performed on the same set of tumor samples that have already been analyzed in NGS (n = 17). The overall concordance between NGS and RT-qPCR data was almost complete with only 5 of 36 comparisons giving discordant statistical testing results (Supplementary Table S2). In all other cases, the direction was identical and scope of change comparable.

### Clinical validation

**Figure 4:**
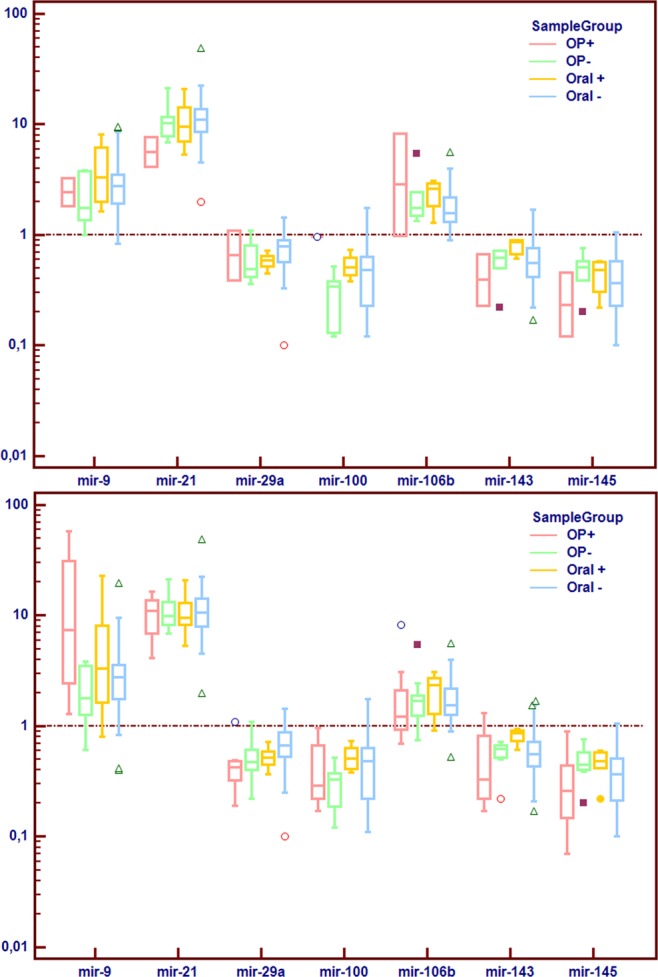
RT-qPCR analysis of selected miRNAs on independent set of clinical samples (top panel) and all clinical samples (bottom panel). Samples were grouped in subgroups based on cancer site and HPV status (based on DNA and RNA positivity). Horizontal referent line is set at fold change 1 and indicates relative expression of control tissue. Median, interquartile range and extreme values are plotted as box and whisker plots, while outliers are indicated as individual markers. HPV = human papillomavirus, SCC = squamous cell carcinoma, OP+ = HPV positive oropharyngeal SCC samples, OP− = HPV negative oropharyngeal SCC samples, Oral+ = HPV positive oral SCC samples, and Oral− = HPV negative oral SCC samples.

Following confirmation of suitable concordance between NGS and qRT-PCR data on the same set of tumor samples, miRs with potential implications in HPV+ HNSCC were selected and their levels assessed on the NGS untested samples (independent set of clinical samples). In addition, the whole set of clinical samples were also assessed (Fig. 4).

To increase robustness, only miRs with at least 100 normalized counts were considered. The following miRs were chosen miR-9-5p, -29a-3p, -106b-5p, 143-3p and -145-5p. Furthermore, generally cancer-related relevant miR-21 and miR-100 were also chosen for this analysis. The analysis reinforced relevance of miR-9 (p = 0.001), miR-21 (p < 0.0001), miR-29a (p = 0.038), miR-100 (p = 0.0029), miR-143 (p = 0.050) and miR-145 (p = 0.006) (Fig. 4) for the HPV+ oropharyngeal subset of the whole set of clinical samples. However, miR-106b was not found to be significantly differently expressed (p = 0.225) in the complete pool of samples.

### Independent validation

Analysis of the public TCGA miRNA sequencing data on comparable set of samples using the same methods also ran into similar obstacles in sample clustering (Supplementary Fig. S2). Ten samples (5 OP+, 1OP−; 1 O+ and 3 O−) were found to behave as outliers and had to be excluded from final analysis. The remaining samples did not cluster according to the sample group when they were analyzed together, however, as before, sub setting the analysis to oral and oropharyngeal subsets improved separations of individual sample sets but was not perfect. Clustering by risk level, which might strengthen the relevant impact of HPV also did not allow clear separation of groups (Supplementary Fig. S2).

We then examined the deregulation of selected miRNAs within TCGA data to examine the concordance of results on two independent data sets. The results of DESeq2 analysis are provided in Supplementary Dataset SD5. Of particular importance, is the miR-9-5p that was confirmed to be significantly upregulated in OP+ subset (log2 fold change of 3.4, adjusted p = 0.0002), while several isomiRs of the same miR were significant in O+ subset. On the other hand, neither mature miR-9 nor its isomiRs were found as significant in OP− or O− subsets. Another very consistent result was the significant upregulation of miR-21-3p in all comparisons; again, indicating the global relevance of this miR. On the other hand, mature miR-29a, -100, -143 and -145 were not found to be significantly deregulated in either set, but their isomiRs were. In the case of OP subset, the direction of change was the same as in our samples and in some cases isomiRs were abundant. Average normalized count of miR-29a isomiR was 3600 (log2FC −1.78, p = 0.012), of miR-100 isomiR was 10000 (log2FC −2.45, p = 3.4E-05), of miR-106b isomiR was 1980 (log2FC 2, p = 0.0001) and of miR-143 isomiR was 56000 (log2 fold change −1.59, p = 0.042). It is also important to note that the deregulation of miR-29a-3p and its isomiR, which was significant in OP+, were however not found to be significantly deregulated in OP− or O− subsets. However, unlike our findings, the same miRNA was not significantly deregulated in O+ subset of TCGA data. The only miRNA that we identified in our samples that couldn’t be confirmed in the reanalysis of TCGA data was miR-27a-5p, which was not found to be significantly deregulated in the TCGA data in any of the comparisons on the mature or any of the isomiR forms.

### miRNA classifiers

The multinomial sparse group lasso method was employed to define minimal set of miRNA sequences capable of correctly classifying samples in defined groups; either based on overall sample group, only HPV status or Ang *et al*.^26^ risk levels (complete or without intermediate risk samples). Twelve different classification models were created (Supplementary Table S3); however, neither model reached high accuracy with the best models correctly estimating only approximately 60% of cases.

### Pathway analysis

To evaluate potential role of miRNA significantly differently expressed exclusively in HPV+ subsets of HNSCC, all such miRs (including isomiRs) with at least 100 normalized counts on average were entered in Diana tools miRPath (v3.0)^32^. IsomiRs were renamed to their parent miRNA and all duplicates were removed. The analysis indicated that at least 62 KEGG (Kyoto encyclopedia of Genes and Genomes)^33^ pathways appear to be significantly associated with miRNA targeted genes. This gene list was based on gene-miRNA validated interactions (Supplementary Dataset SD6). Similar analysis of miRNAs specific for HPV− subsets provided a list of 88 associated cell signaling pathways. Table 2 summarizes cancer relevant KEGG pathways with their rank based on statistical significance in HPV positive and HPV negative cancer subsets. Several pathways have shown large differences between HPV positive and HPV negative subsets. For example, “Transcriptional misregulation in cancer” and “Adherens junction” show 2 orders of magnitude p value difference and are more relevant for HPV positive subset. On the other hand, HPV negative subset appears to be more strongly associated with “HIF-1 signaling pathway”, “Ubiquitin mediated proteolysis”, “TGF-beta signaling pathway”, and “Cell cycle pathway” by several orders of magnitude.

**Table 2:** Selected cancer relevant KEGG pathways significantly associated with HPV positive and HPV negative sample subsets.

KEGG* pathway	HPV positive		HPV negative	
	Rank	p-value	Rank	p-value
Proteoglycans in cancer	1	9.40E-12	1	2.34E-16
Viral carcinogenesis	2	7.90E-08	5	2.88E-09
Hippo signaling pathway	3	1.10E-07	8	6.53E-07
Endocytosis	4	1.36E-07	14	2.07E-06
Transcriptional misregulation in cancer	5	1.36E-07	28	2.62E-05
Pancreatic cancer	6	1.36E-07	25	1.08E-05
Adherens junction	9	2.14E-07	26	1.66E-05
Colorectal cancer	10	2.79E-07	22	5.99E-06
N-Glycan biosynthesis	11	7.31E-07	55	0.003471
Prion diseases	13	8.17E-07	37	0.000605
Pathways in cancer	14	8.17E-07	7	9.2E-08
Glioma	15	1.51E-06	24	9.26E-06
Chronic myeloid leukemia	16	1.87E-06	23	5.99E-06
Cell cycle	18	2.59E-06	2	5.18E-11
ECM-receptor interaction	19	3.19E-06	13	1.71E-06
Renal cell carcinoma	20	8.47E-06	6	2.03E-08
Non-small cell lung cancer	21	1.01E-05	32	0.000182
p53 signaling pathway	22	1.43E-05	20	4.57E-06
Focal adhesion	23	1.63E-05	21	4.57E-06
Protein processing in endoplasmic reticulum	24	2.42E-05	3	9.79E-11
Central carbon metabolism in cancer	26	8.55E-05	42	0.001893
Prostate cancer	28	0.000109	10	1.11E-06
Other types of O-glycan biosynthesis	29	0.00015	49	0.002373
Regulation of actin cytoskeleton	31	0.000257	NA	NA
DNA replication	33	0.00037	43	0.001985
Melanoma	34	0.000472	59	0.006071
Bladder cancer	35	0.000472	57	0.004419
Estrogen signaling pathway	36	0.000491	60	0.006203
Endometrial cancer	37	0.000604	27	2.62E-05
Small cell lung cancer	38	0.000635	19	3.57E-06
Ubiquitin mediated proteolysis	40	0.001667	4	5.33E-10
Thyroid cancer	41	0.002487	45	0.001985
FoxO signaling pathway	42	0.002915	35	0.00043
MAPK signaling pathway	45	0.004836	82	0.040009
Thyroid hormone signaling pathway	47	0.00632	11	1.11E-06
TGF-beta signaling pathway	48	0.00659	12	1.34E-06
Acute myeloid leukemia	50	0.007006	15	3.09E-06
mTOR signaling pathway	52	0.009948	39	0.000605
PI3K-Akt signaling pathway	53	0.018919	71	0.017726
RNA transport	56	0.02175	29	6.04E-05
TNF signaling pathway	57	0.024399	51	0.002373
HIF-1 signaling pathway	61	0.031673	16	3.15E-06

KEGG = Kyoto encyclopedia of Genes and Genomes.

## Discussion

The literature regarding miRNA expression in HNSCC is significant. However, there is no clear consensus on significance of individual miRNA sequences found as deregulated. Specifically, while there are strong overlaps in literature for some miRs (i.e. miR-21, -100, -145) it is difficult to pinpoint a miRNA sequence or set of sequences that would distinguish HPV positive and HPV negative HNSCC to help differentiate those two etiologically different subtypes of HNSCC. In most cases, potential miRNAs are found equally often by studies focusing on HPV positive but also by studies focusing on HPV negative HNSCC reducing the chance that deregulation of such a miRNA is HPV related. To add to the current knowledge, we assessed the miR profiles in a well described set of clinical HNSCC samples. The study group consisted of primary oral and oropharyngeal tumors with known HPV status on both DNA and RNA level. The overall HPV prevalence (DNA and RNA positive) was 23% but that increased in tonsil subset (57%). HPV prevalence and overall structure of patients fits well with current literature on HNSCC^34^. However, it has to be noted that median ages of HPV positive and HPV negative groups overall were similar, although in general HPV related HNSCC are usually more associated with lower age^10^.

It is still unclear why miRNA profiles exhibit such large differences among studies. One of the likely reasons is the overall heterogeneity of HNSCC as a group leading different authors to include differently structured sample groups in their studies. Furthermore, unstandardized methods of HPV detection or inference of its presence (DNA, RNA, p16) also led to different groupings if at all considered. Added to that were methodological differences in miRNA detection, quantitation and normalization in each study. However, another less evident reason for discrepancies could be the isomiRs. In our main NGS sequencing experiment significant differential regulation of 1145 miRNA sequences was detected across comparisons but the majority were actually isomiR sequences (Supplementry Dataset S3). It is currently unknown to which extent do these sometimes very abundant forms affect results of other studies where they cannot be distinguished from mature miRNA when utilizing methods other than NGS. On the other hand, several studies have thus far pointed out the independent importance of isomiRs in gene regulation^35–37^, thus the isomiRs cannot be simply disregarded.

Another important factor possibly confounding both previous and current findings is the fact that HPV itself does not need be a causal factor in tumor development even if found in the tumor as other factors like smoking might have a stronger impact. As emphasized in the study by Ang *et al*.^26^, three distinct survival profiles were observed. The greatest risk was for HPV negative OPSCC patients, however, HPV positive patients were in the lowest risk group only if they didn’t smoke or had tumors with lower nodal stage. It is possible that inclusion of samples classified as intermediate risk group might confound miRNA results and associations both herein and in other previous studies. Indeed, 2 of our 9 sequenced HPV positive samples could be classified as intermediate risk group, where in addition to HPV, other factors like smoking might further influence the miRNA profiles. However, classifying samples only according to Ang *et al*.^26^ risk factors still did not resolve the issue of suboptimal separation either in our samples or those in TCGA.

Another outcome of the miRNA NGS profiling was the apparent inability of this method to completely differentiate 4 specific subgroups of samples (Figs. 1 and 2); only control samples could be resolved clearly. Better separation of samples in OPSCC group (Fig. 2B) is possibly due to larger influence of HPV at oropharyngeal site noting that HPV is known to be less relevant for the development of oral cancer. Another interesting observation (but with very limited number of samples) is that samples positive for the unspliced form of HPV16-E6 mRNA clustered close to HPV negative samples (Fig. 2B), implying that HPV is also less etiologically relevant in that case. Previously transcriptionally negative HNSCC were also shown to have survival similar to HPV negative HNSCC^38^. While it is possible that the detected form of unspliced mRNA is due to DNA carryover, this is unlikely as RNAse-free DNAse step during RNA isolation was performed to minimize such possibility. It is also interesting to note that this sample was classified as an intermediate risk group sample (Supplementary Fig. S1), again implying that other factors could be confounding HPV activity.

To verify the validity of NGS data, selected miRNA sequences were assessed on both the same samples (technical) and all clinical samples by qRT-PCR. The concordance of two methods on the same samples was very good (Supplementary Table S2); hence, validating the reliability of our results. The NGS results indicated the relevance of 16 mature miRNAs (miR-9-5p, 25-5p, -29a-3p, -29b-3p, -34a-5p, -93-5p, 106b-5p, -133a-5p, -133a-3p, -139-5p, -140-5p, -147b, -208b-3p, 210-5p, 328-3p, -1307-3p) for HPV positive oropharyngeal subset. Analyzing a further selection of potentially relevant miRs on the whole set of samples reinforced the relevance of miR-9 (p = 0.001) and miR-29a (p = 0.038) for HPV+ OPSCC. Results also reinforced the overall relevance of miR-21 (p < 0.0001 in OP+) and miR-100 (p < 0.0029 in OP+). However, in this study, as well as in the literature, those miRNAs are also associated with HPV negative tumors and thus are unlikely to be HPV associated.

Another validation was performed by reanalyzing miRNA sequencing data of a completely independent set of HNSCC cancer cases obtained from the publicly available TCGA portal. The analysis of this set of samples has also shown that miRNA sequencing cannot readily separate sample clusters, but somewhat better separation can be seen when oral and oropharyngeal subsets are analyzed separately (Supplementary Fig. S2). It is very important to highlight that miR-9 was found to be significantly upregulated in TCGA OP+ subset, its isomiRs in O+ subset, and was completely absent from HPV negative subsets. Another completely concordant result was for miR-21-3p, which was found significantly upregulated in all comparisons. Furthermore, other highlighted miRNAs selected for clinical set validation (-29a, -100, -106b, -143 and -145) were found to be significantly deregulated in the same direction at the isomiR level in the OP+ subset. It is important to note that our data and the data of TCGA was analyzed starting from raw count data, which was imported to R for DESeq2 analysis. However, there were some methodological differences up to that point, which might influence subsequent results. Namely, we used Illumina Basespace and SmallRNA app for alignment and counting, while TCGA data was aligned, counted and isomiRs presented differently. Despite that, results were highly comparable on clustering based on global miRNA profiles as well as deregulation of specific miRNA sequences. Reanalysis of TCGA data also indicates that miR-9 and -29a are relevant in OP+ subset and miR-9 is also relevant in O+ subset.

The expression of both miR-9 and miR-29a have previously been found deregulated in HNSCC (Supplementary Dataset SD2). Furthermore, both miRNAs were thoroughly reviewed very recently in the context of different cancer types^39,40^. Also, a similar systematic review of miRNA in cervical cancer indicated both miR-9 and miR-29 as consistently deregulated in literature and relevant in cervical cancer development^41^.

Briefly, miR-9 appears to be upregulated by HPV-E6 and when upregulated, it blocks keratinocyte differentiation and induces proliferation and migration^39^. However, its roles are very dependent on the context. Other studies have shown miR-9 to be upregulated in recurrent HNSCC^42^, but also as a potentially good salivary^43^ and even methylation biomarker^44^. It was one of the few overlapping miRs identified between HNSCC and cervical cancer^22^. More importantly, previous functional studies have already shown that miR-9 seems to be the miRNA most activated by HPV E6 protein in cervical cancer^45^.

MiR-29a was also shown to behave differently depending on the context^40^, but it is most often downregulated across cancers, and thus might be considered tumor suppressive. It was shown to influence proliferation, apoptosis, angiogenesis and metastasis depending on cancer type. Interestingly, miR-29a was also shown to induce drug resistance^40^, which might also explain some of the survival differences between HPV positive and HPV negative cancers if it is more often (but not exclusively) affected in HPV positive cancers.

Following examination of individual miRNAs, we also attempted to produce classifiers based on combinations of several miRNA sequences (Supplementary Table S3). A sparse group lasso method was employed with samples grouped according to different criteria (overall sample group, HPV status only and Ang *et al*.^26^ risk levels). We created different models where classifier was trained either on our data or TCGA data and then used to classify the other set. Since the maximal success rate was 64%, no classifier model was robust enough. Interestingly, models trained on one half of TCGA data and tested on the other half of TCGA data were also not successful even though their cross validation error rates were very low. It appears that large sample heterogeneity, even in the independent sample sets such as TCGA, might in part explain the discrepancies in previous literature. It probably is interesting to note that classifiers based on miRNA expression might need further specialized statistical modeling to decrease the impact of “normal tissue contamination” on the classifier^29^; however, this was beyond the scope of the current study.

The analysis of deregulated miRNAs in HPV+ and HPV− HNSCC using miRpath to assess KEGG pathway associations of functionally confirmed miR target genes revealed the main signaling pathways involved in the disease development (Table 2, Supplementary Dataset SD6). As expected, miRs found in both sets are associated with many other cancer related pathways, however, there were differences in strength of association with the particular pathways. The results indicated that HPV might be more involved with transcriptional dysregulation in cancer and adherens junction pathways, while miRNA profile of HPV− HNSCC implies stronger relevance of HIF-1 and TGF-beta pathways. The adherens junction pathway is of particular interest as high risk HPV types are known to interact and degrade many cell polarity proteins by E6-PDZ domain interactions^46^. It appears that this important viral process is also supported by consequent or parallel miRNA profile changes. In contrast, miRNA profile of HPV− HNSCC suggests stronger associations with more general pathways. These dissimilarities also support different etiologies of HPV positive and negative tumors.

In summary, miRNA landscape of HNSCC is very heterogeneous, primarily due to heterogeneity of sample material (and miRNA abundance therein), different methods (of miRNA detection, isomiR inclusion, HPV determination) and different grouping of samples during analysis (or lack of subgroup separation and even biological importance of HPV in cancer development). Despite this, some miRNAs show consistency, in particular miRs -21, -100 and -145 are overall relatively consistently detected in all HNSCC studies. However, of HPV specific miRs, only miR-9 seems to be consistently found in HPV positive and rarely in HPV negative subsets of HNSCC, including results from clinical samples in this study and specifically the analysis of TCGA data. Thus, miR-9 is the most likely miRNA specific for the HNSCC with the HPV etiology. While overall miRNA profiles also show lack of consistency and do not easily allow classification based on specific patterns, it appears that miRNAs identified in HPV positive and HPV negative cancers possibly affect cancer relevant pathways differently hence, reinforcing their etiological differences.

## Supplementary information

Supplementary Information

Dataset 1

Dataset 2

Dataset 3

Dataset 4

Dataset 5

Dataset 6

## Data Availability

Raw sequences of the NGS experiment were uploaded to the ArrayExpress repository with the accession number E-MTAB-7030 (www.ebi.ac.uk/arrayexpress). All data generated or analyzed during this study are included in this published article (and its Supplementary Information files).
