# Supplementary material for: Genome-wide miRNA profiling reinforces the importance of miR-9 in human papillomavirus associated oral and oropharyngeal head and neck cancer
Source: Sci Rep. 2019 Feb 19;9:2306. doi: 10.1038/s41598-019-38797-z (PMC6381209; doi:10.1038/s41598-019-38797-z)
Supplement: Supplementary file 1 — Supplementary Information [file 41598_2019_38797_MOESM1_ESM.docx]

## Genome-wide miRNA profiling reinforces the importance of miR-9 in human papillomavirus associated oral and oropharyngeal head and neck cancer

Ksenija Božinović, Ivan Sabol, Emil Dediol, Nina Milutin Gašperov, Spomenka Manojlović, Zuzana Vojtechova, Ruth Tachezy, Magdalena Grce

## **Supplementary Literature**

Previous reviews have already hinted at the significant discrepany of miRNA findings across studies. Therefore, studies focused on high and medium throughput analysis of miRNA in the head and neck cancer (HNSCC) were searched within NCBI Pubmed database. Studies were accessed and wherever possible raw or supplemental data including all significantly deregulated miRNAs were extracted and tabulated. Where the information regarding -5' or -3' strand was not given, we considered the miRNA to belong to the more abundant form acording to miRbase. If a miRNA was marked with „*“ we considered it to correspond to less abundant form according to miRbase. Additionally, included articles as well as published reviews touching on the topic were screened for additional references to primary publications but reviews themselves were not included unless summarizing primarily low throughput studies. Thus care has been taken to include each primary study only once within the final table. The exception was when a particular study presented the differential expression results of several comparisons in which case significantly deregulated miRNAs from each comparison are listed as a separate column. Additionally, studies examining miRNA deregulation in cervical cancer but included in the HNSCC focused reviews were also included to allow cross tissue examination of HPV relevant miRNAs.

In this way the miRNA data from a total of 62 studies were included in the final comparison (Supplemental table 7). Of those, 11 were examining head and neck sites in a HPV positive context and 11 in HPV negative context. On the other hand majority of studies (n=36) did not consider HPV status in any way. Aditionally there were 11 studies investigting cervical or foreskin keratinocytes. Final 3 included studies presented reviews or conceptual models without patient material data.

1. Avissar 2009 ^1^
2. Bonnin 2016 ^2^
3. Bose 2013 ^3^
4. Cervigne 2009 ^4^
5. Chang 2008 ^5^
6. Chen 2012 ^6^
7. Chen 2018 ^7^
8. Chiantore 2016 ^8^
9. Childs 2009 ^9^
10. de Carvalho 2015 ^10^
11. Dreher 2011 ^11^
12. Fukumoto 2014 ^12^
13. Fukumoto 2015 ^13^
14. Ganci Federica 2015 ^14^
15. Gao 2013 ^15^
16. Greco 2011 ^16^
17. He 2016 ^17^
18. Hu 2010 ^18^
19. Hui 2010 ^19^
20. Hui 2013 ^20^
21. Jung 2012 ^21^
22. Kikkawa 2010 ^22^
23. Koshizuka 2017 ^23^
24. Lajer 2011 ^24^
25. Lajer 2012 ^25^
26. Langevin 2017 ^26^
27. Li 2009 ^27^
28. Li 2011 ^28^
29. Lindenbergh-van der Plas 2013 ^29^
30. Liu 2010 ^30^
31. Lui 2007 ^31^
32. Manasa and Kannan 2017 ^32^
33. Manikandan 2016 ^33^
34. Martinez 2008 ^34^
35. Martinez 2015 ^35^
36. Miller 2015 ^36^
37. Nair 2015 ^37^
38. Nohata 2011 ^38^
39. Nunez Lopez 2018 ^39^
40. Quabius 2017 ^40^
41. Ramdas 2009 ^41^
42. Rao 2012 ^42^
43. Salazar 2014 ^43^
44. Sannigrahi 2018 ^44^
45. Scapoli 2010 ^45^
46. Schneider 2018 ^46^
47. Severino 2013 ^47^
48. Severino 2013 ^48^
49. Vojtechova 2016 ^49^
50. Wald 2011 ^50^
51. Wan 2017 ^51^
52. Wang 2008 ^52^
53. Wang 2014 ^53^
54. Wang 2016 ^54^
55. Wong 2008 ^55^
56. Wong 2016 ^56^
57. Xu 2015 ^57^
58. Yan 2017 ^58^
59. Zhang 2014 ^59^
60. Zhang 2016 ^60^
61. Zhou 2016 ^61^
62. Zou 2016 ^62^

**Supplementary Table S1.** Sample annotation for samples selected for NGS miRNA sequencing analysis

| **Index** | **Outlier** | **ID** | **Sample**  **Group** | **Tissue** | **Region** | **HPV** | **RNA** | **Tumor** | **Reads** | **Gender** | **Age** | **T** | **N** | **Stage 7th ed** | **Ang risk level** | **Smoking/**  **Drinking** | **Gradus** |
| --- | --- | --- | --- | --- | --- | --- | --- | --- | --- | --- | --- | --- | --- | --- | --- | --- | --- |
| 1 |  | KBD3 | O+ | floor | oral | 1 | 2 | SCC | 7839962 | M | 59 | 4a | 0 | IVa | Low | 1 | 2 |
| 2 |  | KBD10 | O- | floor | oral | 0 | 0 | SCC | 8923969 | M | 61 | 2 | 0 | II | High | 1 | 2 |
| 3 |  | KBD15 | OP+ | tonsil | oropharyngeal | 1 | 2 | SCC | 10939863 | M | 66 | 4a | 1 | IVa | Low | 1 | 2 |
| 4 |  | KBD17 | OP+ | tonsil | oropharyngeal | 1 | 2 | SCC | 1706510 | F | 32 | 1 | 1 | III | Low | 0 | ? |
| 5 |  | KBD1c | O- | floor | oral | 0 | 0 | SCC | 1507508 | M | 66 | 3 | 2b | IVa | High | 1 | 2 |
| 6 |  | KBD2 | O- | floor | oral | 0 | 0 | SCC | 3599588 | M | 61 | 2 | 1 | III | High | 1 | 2 |
| 7 |  | KBD4 | OP- | tonsil | oropharyngeal | 0 | 0 | SCC | 2446352 | M | 53 | 4a | 3 | IVb | High | 1 | ? |
| 8 |  | KBD6 | O- | floor | oral | 0 | 0 | SCC | 2618421 | M | 52 | 2 | 0 | II | High | 1 | 2 |
| 9 | Y | KBD9 | O- | floor | oral | 0 | 0 | SCC | 2048688 | M | 53 | 4a | 0 | IVa | High | 1 | 1 |
| 10 |  | KBD11 | OP- | base of tongue | oropharyngeal | 0 | 0 | SCC | 916069 | F | 55 | 3 | 1 | III | High | 1 | 1 |
| 11 |  | KBD13 | O+ | tongue | oral | 1 | 1 | SCC | 3305320 | M | 31 | 4a | 2b | IVa | Low | 0 | 1 |
| 12 |  | KBD16 | OP- | tonsil | oropharyngeal | 0 | 0 | SCC | 2622547 | M | 56 | 4a | 1 | IVa | High | 1 | 2 |
| 13 |  | KBD18 | O- | floor | oral | 0 | 0 | SCC | 8088982 | M | 56 | 2 | 1 | III | High | 1 | 1 |
| 14 |  | KBD19 | OP+ | base of tongue | oropharyngeal | 1 | 1 | SCC | 2745721 | M | 64 | 3 | 3 | IVb | Intermed | 1 | 2 |
| 15 |  | KBD21 | O+ | tongue | oral | 1 | 1 | SCC | 2086376 | M | 69 | 1 | 2 | IVa | Low | 1 | 3 |
| 16 | Y | KBD25 | OP- | base of tongue | oropharyngeal | 0 | 0 | SCC | 1951506 | F | 57 | 3 | 2 | IVa | High | 1 | 2 |
| 17 |  | RNA3T | N | tonsil | oropharyngeal | 0 | 0 | normal | 3583573 | F | 59 | 0 | 0 | 0 | N | 1 | 0 |
| 18 |  | RNA10T | N | tonsil | oropharyngeal | 0 | 0 | normal | 281857 | F | 59 | 0 | 0 | 0 | N | 1 | 0 |
| 19 |  | RNA16T | N | tonsil | oropharyngeal | 0 | 0 | normal | 1231446 | F | 59 | 0 | 0 | 0 | N | 1 | 0 |
| 20 |  | ORL224 | OP+ | tonsil | oropharyngeal | 1 | 2 | SCC | 3456222 | F | 73 | 3 | 3 | IVb | Low | 0 | 2 |
| 21 |  | ORL116 | OP+ | tonsil | oropharyngeal | 1 | 2 | SCC | 1817979 | M | 59 | 3 | 3 | IVb | Intermed | 1 | 2 |
| 22 |  | ORL161 | OP+ | tonsil | oropharyngeal | 1 | 2 | SCC | 2921036 | M | 55 | 3 | 2 | IVa | Low | 1 | 3 |

* RNA 0 means negative or not tested; RNA 1 means full length of E6 RNA detected; RNA 2 means spliced form of E6 RNA detected

**Supplementary figure S1**. Sample clustering on Principal component analysis (PCA) of all miRNA sequences (including isomirs) on A) all samples, or only oropharyngeal or oral subsets (B and C respectively). Samples are classified according to the risk level as suggested by Ang et al.


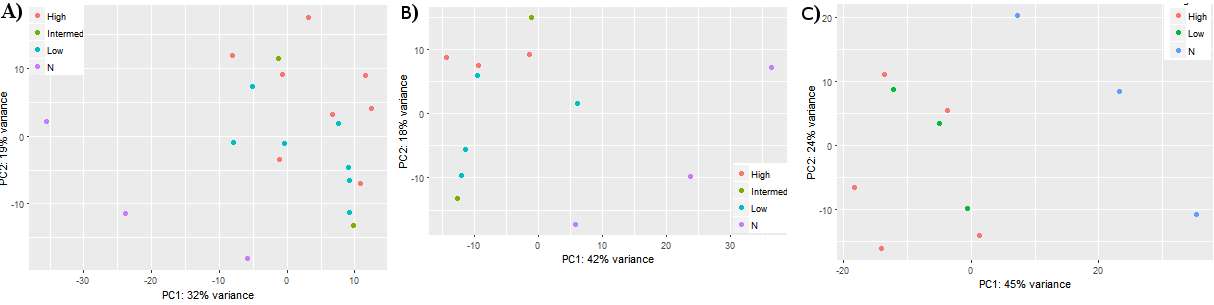


**Supplementary Table S2**. Technical validation results of miRNA sequencing and qRT-PCR methods performed on the identical set of samples. Very high concordance of results obtained by both methods both in the direction of deregulation and its significance can be seen (highlighted in green) across almost all comparisons.

| miRNA | literature data | NGS Fold change and p value * | | | | | | | | qPCR Fold change and p value * | | | | | | | |
| --- | --- | --- | --- | --- | --- | --- | --- | --- | --- | --- | --- | --- | --- | --- | --- | --- | --- |
|  |  | OP+ | | OP- | | O+ | | O- | | OP+ | | OP- | | O+ | | O- | |
|  |  | FC | p | FC | p | FC | p | FC | p | FC | p | FC | p | FC | p | FC | p |
| hsa-miR-21-3p | upregulated across cancer | 10.061 | 0.000 | 11.847 | 0.000 | 6.767 | 0.000 | 4.395 | 0.044 | 11.454 | 0.000 | 11.799 | 0.001 | 9.233 | 0.000 | 10.476 | 0.001 |
| hsa-miR-31-5p | upregulated across cancer | 11.433 | 0.000 | 20.920 | 0.000 | 8.383 | 0.000 | 17.575 | 0.000 | 10.675 | 0.002 | 23.847 | 0.049 | 13.673 | 0.016 | 27.578 | 0.000 |
| hsa-miR-100-5p | downregulated across cancer | -2.739 | 0.060 | -2.795 | 0.022 | -1.919 | 0.339 | -1.169 | 0.853 | -3.278 | 0.005 | -3.433 | 0.005 | -3.227 | 0.124 | -3.019 | 0.139 |
| hsa-miR-34a-5p | upregulated | 2.444 | 0.000 | 1.357 | 0.591 | 1.451 | 0.590 | 1.178 | 0.721 | 4.093 | 0.000 | 1.698 | 0.274 | 2.918 | 0.018 | 1.931 | 0.221 |
| hsa-miR-27a-5p |  | 4.546 | 0.006 | 4.221 | 0.018 | 5.106 | 0.002 | 7.080 | 0.000 | 8.632 | 0.000 | 7.052 | 0.005 | 10.682 | 0.001 | 14.702 | 0.002 |
| hsa-miR-9-5p |  | 12.793 | 0.000 | 1.533 | 0.574 | 9.241 | 0.002 | 1.706 | 0.474 | 12.545 | 0.002 | 1.642 | 0.516 | 6.085 | 0.234 | 1.243 | 0.809 |
| hsa-miR-143-3p | downregulated | -2.369 | 0.110 | -1.730 | 0.265 | -2.320 | 0.129 | -3.111 | 0.039 | -2.410 | 0.068 | -1.705 | 0.192 | -1.293 | 0.323 | -2.123 | 0.276 |
| hsa-miR-145-5p |  | -4.277 | 0.013 | -3.253 | 0.038 | -3.351 | 0.070 | -4.224 | 0.006 | -3.828 | 0.013 | -2.195 | 0.106 | -3.111 | 0.107 | -3.675 | 0.089 |
| hsa-miR-218-5p |  | -2.624 | 0.053 | -1.767 | 0.399 | -1.991 | 0.374 | -1.945 | 0.255 | -3.225 | 0.014 | -1.818 | 0.201 | -2.455 | 0.317 | -1.621 | 0.440 |
| hsa-miR-16-5p | manufacturer ref | 1.470 | 0.179 | 1.560 | 0.283 | 1.408 | 0.540 | 1.263 | 0.532 | referent | | | | | | | |
| hsa-miR-191-5p | manufacturer ref | -1.161 | 0.715 | 1.203 | 0.737 | 1.146 | 0.861 | 1.054 | 0.925 |  |  |  |  |  |  |  |  |
| hsa-miR-181a-5p | consistently expressed in NGS | -1.066 | 0.914 | -1.304 | 0.563 | -1.011 | 0.991 | -1.257 | 0.594 |  |  |  |  |  |  |  |  |

*green cells indicate agreement of NGS results and qPCR results in both direction of deregulation and p value significance; non significant p values are printed in grey as are fold changes less than 1,3 in either direction

**Supplementary Figure S2**. TCGA Sample clustering on Principal Component Analysis (PCA) of all miRNA sequences on A) all samples with outliers. B) and C) present heatmap or PCA clustering of retained samples based on all miRNA sequences. D) clustering of all retained samples grouped by Ang risk groups. Panels E-H present oropharyngeal (E/G) or oral (F/H) subsets labeled by either sample (E/F) or risk (G/H) groups. Neither strategy allows optimal clustering of samples.


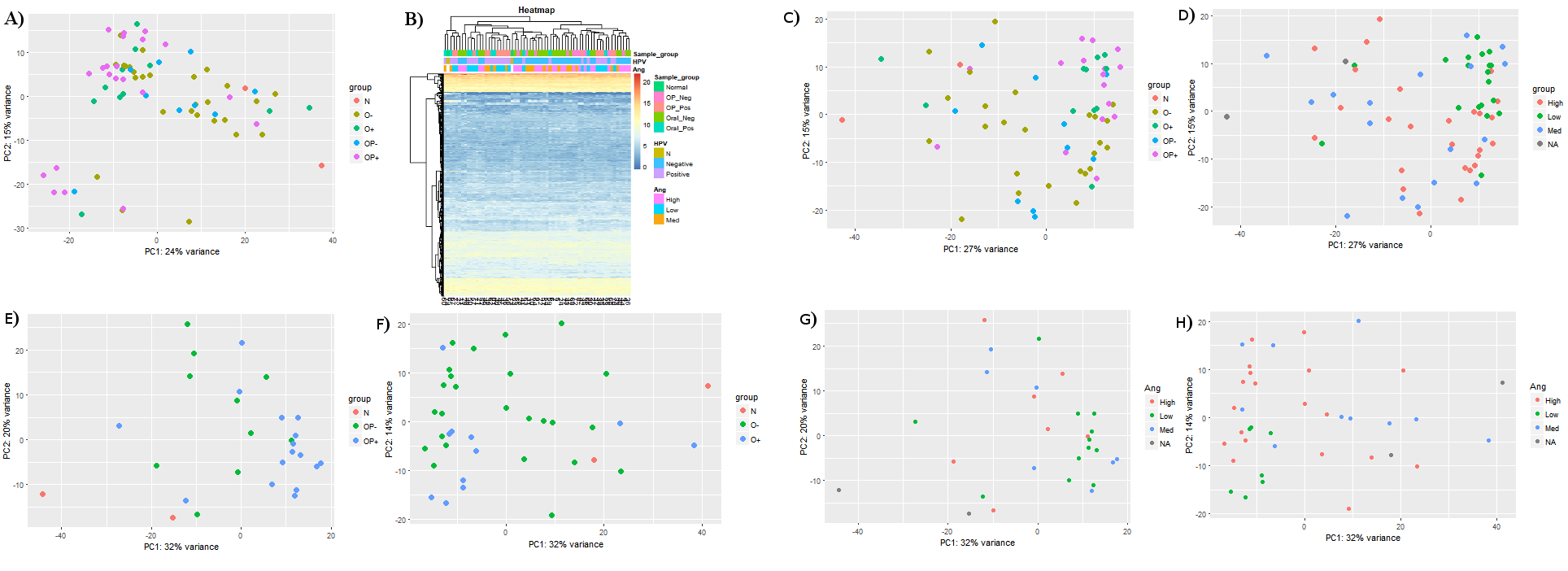


**Supplementary Table S3**. Different miRNA classifiers prepared with multinomial sparse group lasso method for classification of HNSCC cases. Classifiers were trained on one part of data and the model tested on another part of data.

| **Classifier (sample groups)** | **Training set** | **Test set** | **Cross validation error rate** | **Correct predictions** | **Features included in model** |
| --- | --- | --- | --- | --- | --- |
| Location & HPV (OP+, OP-, O+, O-) | author data | TCGA data | 0.71 | 15.30% | hsa-miR-34a-5p |
| Location & HPV (OP+, OP-, O+, O-) | TCGA data | author data | 0.4 | 41.20% | hsa-let-7i-3p, hsa-miR-101-3p, hsa-miR-106a-5p, hsa-miR-125b-5p, hsa-miR-146a-5p, hsa-miR-15b-5p, hsa-miR-17-3p, hsa-miR-17-5p, hsa-miR-200a-5p, hsa-miR-200b-3p, hsa-miR-200b-5p, hsa-miR-200c-3p, hsa-miR-23b-3p, hsa-miR-27b-3p, hsa-miR-30e-5p, hsa-miR-429, hsa-miR-590-3p, hsa-miR-625-3p, hsa-miR-625-5p, hsa-miR-654-3p, hsa-miR-769-5p, hsa-miR-944, |
| Location & HPV (OP+, OP-, O+, O-) | 1/2 TCGA data | 1/2 TCGA data | 0.56 | 13.90% | hsa-let-7b-5p, hsa-let-7i-3p, hsa-miR-132-5p, hsa-miR-143-5p, hsa-miR-144-5p, hsa-miR-15b-5p, hsa-miR-185-5p, hsa-miR-193b-3p, hsa-miR-200b-3p, hsa-miR-200b-5p, hsa-miR-200c-3p, hsa-miR-203a-3p, hsa-miR-210-3p, hsa-miR-22-3p, hsa-miR-223-3p, hsa-miR-27a-5p, hsa-miR-27b-3p, hsa-miR-28-5p, hsa-miR-301a-5p, hsa-miR-30b-5p, hsa-miR-339-3p, hsa-miR-340-3p, hsa-miR-382-3p, hsa-miR-429, hsa-miR-451a, hsa-miR-582-3p, hsa-miR-769-5p, hsa-miR-889-3p, |
| HPV only (HPV+, HPV-) | author data | TCGA data | 0.59 | 62.50% | hsa-let-7a-5p, hsa-let-7e-5p, hsa-miR-10b-5p, hsa-miR-127-3p, hsa-miR-181b-5p, hsa-miR-223-3p, hsa-miR-28-3p, hsa-miR-30c-5p, hsa-miR-30e-5p, hsa-miR-33a-5p, hsa-miR-582-3p, hsa-miR-9-5p, |
| HPV only (HPV+, HPV-) | TCGA data | author data | 0.11 | 52.90% | hsa-let-7i-3p, hsa-miR-15b-5p, hsa-miR-181a-2-3p, hsa-miR-181b-5p, hsa-miR-183-5p, hsa-miR-200a-5p, hsa-miR-200b-3p, hsa-miR-23b-3p, hsa-miR-26a-5p, hsa-miR-27b-3p, hsa-miR-30a-3p, hsa-miR-365a-3p, hsa-miR-365b-3p, hsa-miR-378a-3p, |
| HPV only (HPV+, HPV-) | 1/2 TCGA data | 1/2 TCGA data | 0.17 | 63.90% | hsa-let-7i-3p, hsa-miR-15b-3p, hsa-miR-15b-5p, hsa-miR-200b-3p, hsa-miR-23b-3p, hsa-miR-30a-3p, hsa-miR-378a-3p, |
| Ang risk (High, Medium, Low) | author data | TCGA data | 0.53 | 47.20% | hsa-let-7a-5p, hsa-let-7e-5p, hsa-miR-103a-3p, hsa-miR-10b-5p, hsa-miR-127-3p, hsa-miR-183-5p, hsa-miR-223-3p, hsa-miR-28-3p, hsa-miR-30a-3p, hsa-miR-30c-5p, hsa-miR-30e-5p, hsa-miR-33a-5p, hsa-miR-342-3p, hsa-miR-484, hsa-miR-582-3p, hsa-miR-9-5p, |
| Ang risk (High, Medium, Low) | TCGA data | author data | 0.33 | 11.80% | hsa-let-7b-5p, hsa-let-7d-3p, hsa-let-7i-3p, hsa-miR-133a-3p, hsa-miR-143-3p, hsa-miR-155-5p, hsa-miR-15b-5p, hsa-miR-181a-5p, hsa-miR-183-5p, hsa-miR-200a-5p, hsa-miR-200b-3p, hsa-miR-23b-3p, hsa-miR-24-3p, hsa-miR-26a-5p, hsa-miR-27b-3p, hsa-miR-28-5p, hsa-miR-30a-3p, hsa-miR-30c-5p, hsa-miR-365a-3p, hsa-miR-365b-3p, hsa-miR-378a-3p, hsa-miR-429, hsa-miR-486-5p, hsa-miR-99b-5p, |
| Ang risk (High, Medium, Low) | 1/2 TCGA data | 1/2 TCGA data | 0.31 | 50.00% | hsa-let-7i-3p, hsa-miR-1307-3p, hsa-miR-200b-3p, hsa-miR-28-5p, hsa-miR-30c-5p, hsa-miR-33a-5p, hsa-miR-429, |
| Ang extreme groups (High, Low) | author data | TCGA data | 0.67 | 60.00% | hsa-miR-106a-5p, hsa-miR-132-5p, hsa-miR-155-5p, hsa-miR-183-5p, hsa-miR-223-3p, hsa-miR-550a-3p, hsa-miR-625-5p, hsa-miR-9-3p, |
| Ang extreme groups (High, Low) | TCGA data | author data | 0.13 | 46.70% | hsa-let-7i-3p, hsa-miR-106a-5p, hsa-miR-15b-5p, hsa-miR-181b-2-3p, hsa-miR-183-5p, hsa-miR-193b-3p, hsa-miR-200a-5p, hsa-miR-200b-3p, hsa-miR-200c-5p, hsa-miR-23b-3p, hsa-miR-27b-3p, hsa-miR-3065-3p, hsa-miR-30a-3p, hsa-miR-365a-3p, hsa-miR-365b-3p, hsa-miR-3677-3p, hsa-miR-378a-3p, hsa-miR-378a-5p, hsa-miR-379-5p, hsa-miR-429, hsa-miR-7-5p, |
| Ang extreme groups (High, Low) | 1/2 TCGA data | 1/2 TCGA data | 0.08 | 63.30% | hsa-let-7i-3p, hsa-miR-132-5p, hsa-miR-144-5p, hsa-miR-154-5p, hsa-miR-15b-3p, hsa-miR-15b-5p, hsa-miR-200b-3p, hsa-miR-331-3p, hsa-miR-429, |

## **Supplementary references**

1. Avissar, M., Christensen, B. C., Kelsey, K. T. & Marsit, C. J. A MicroRNA Expression Ratio is Predictive of Head and Neck Squamous Cell Carcinoma. *Clin. Cancer Res. Off. J. Am. Assoc. Cancer Res.* **15**, 2850–2855 (2009).

2. Bonnin, N. *et al.* MiR-422a promotes loco-regional recurrence by targeting NT5E/CD73 in head and neck squamous cell carcinoma. *Oncotarget* **7**, 44023–44038 (2016).

3. Bose, P., Brockton, N. T. & Dort, J. C. Head and neck cancer: from anatomy to biology. *Int. J. Cancer* **133**, 2013–2023 (2013).

4. Cervigne, N. K. *et al.* Identification of a microRNA signature associated with progression of leukoplakia to oral carcinoma. *Hum. Mol. Genet.* **18**, 4818–4829 (2009).

5. Chang, S. S. *et al.* MicroRNA alterations in Head and Neck Squamous Cell Carcinoma. *Int. J. Cancer J. Int. Cancer* **123**, 2791–2797 (2008).

6. Chen, Z. *et al.* Down-regulation of the microRNA-99 family members in head and neck squamous cell carcinoma. *Oral Oncol.* **48**, 686–691 (2012).

7. Chen, L. *et al.* Prediction of radiotherapy response with a 5‐microRNA signature‐based nomogram in head and neck squamous cell carcinoma. *Cancer Med.* **7**, 726–735 (2018).

8. Chiantore, M. V. *et al.* Human papillomavirus E6 and E7 oncoproteins affect the expression of cancer-related microRNAs: additional evidence in HPV-induced tumorigenesis. *J. Cancer Res. Clin. Oncol.* **142**, 1751–1763 (2016).

9. Childs, G. *et al.* Low-Level Expression of MicroRNAs let-7d and miR-205 Are Prognostic Markers of Head and Neck Squamous Cell Carcinoma. *Am. J. Pathol.* **174**, 736–745 (2009).

10. de Carvalho, A. C. *et al.* Accuracy of microRNAs as markers for the detection of neck lymph node metastases in patients with head and neck squamous cell carcinoma. *BMC Med.* **13**, (2015).

11. Dreher, A. *et al.* Differential expression of cellular microRNAs in HPV 11, -16, and -45 transfected cells. *Biochem. Biophys. Res. Commun.* **412**, 20–25 (2011).

12. Fukumoto, I. *et al.* Identification of tumour suppressive microRNA-451a in hypopharyngeal squamous cell carcinoma based on microRNA expression signature. *Br. J. Cancer* **111**, 386–394 (2014).

13. Fukumoto, I. *et al.* MicroRNA expression signature of oral squamous cell carcinoma: functional role of microRNA-26a/b in the modulation of novel cancer pathways. *Br. J. Cancer* **112**, 891–900 (2015).

14. Ganci Federica *et al.* MicroRNA expression as predictor of local recurrence risk in oral squamous cell carcinoma. *Head Neck* **38**, E189–E197 (2015).

15. Gao, G. *et al.* A microRNA expression signature for the prognosis of oropharyngeal squamous cell carcinoma. *Cancer* **119**, 72–80 (2013).

16. Greco, D. *et al.* Human Papillomavirus 16 E5 Modulates the Expression of Host MicroRNAs. *PloS One* **6**, e21646 (2011).

17. He, Q. *et al.* MicroRNA-21 regulates prostaglandin E2 signaling pathway by targeting 15-hydroxyprostaglandin dehydrogenase in tongue squamous cell carcinoma. *BMC Cancer* **16**, (2016).

18. Hu, X. *et al.* A MicroRNA Expression Signature for Cervical Cancer Prognosis. *Cancer Res.* **70**, 1441–1448 (2010).

19. Hui, A. B. Y. *et al.* Comprehensive MicroRNA Profiling for Head and Neck Squamous Cell Carcinomas. *Clin. Cancer Res.* **16**, 1129–1139 (2010).

20. Hui, A. B. Y. *et al.* Potentially prognostic miRNAs in HPV-associated oropharyngeal carcinoma. *Clin. Cancer Res.* **19**, 2154–2162 (2013).

21. Jung, H. M. *et al.* Keratinization-associated miR-7 and miR-21 Regulate Tumor Suppressor Reversion-inducing Cysteine-rich Protein with Kazal Motifs (RECK) in Oral Cancer. *J. Biol. Chem.* **287**, 29261–29272 (2012).

22. Kikkawa, N. *et al.* miR-489 is a tumour-suppressive miRNA target PTPN11 in hypopharyngeal squamous cell carcinoma (HSCC). *Br. J. Cancer* **103**, 877–884 (2010).

23. Koshizuka, K. *et al.* Deep sequencing-based microRNA expression signatures in head and neck squamous cell carcinoma: dual strands of pre-miR-150 as antitumor miRNAs. *Oncotarget* **8**, 30288–30304 (2017).

24. Lajer, C. B. *et al.* Different miRNA signatures of oral and pharyngeal squamous cell carcinomas: a prospective translational study. *Br. J. Cancer* **104**, 830–840 (2011).

25. Lajer, C. B. *et al.* The role of miRNAs in human papilloma virus (HPV)-associated cancers: bridging between HPV-related head and neck cancer and cervical cancer. *Br. J. Cancer* **106**, 1526–1534 (2012).

26. Langevin, S. *et al.* Comprehensive microRNA-sequencing of exosomes derived from head and neck carcinoma cells in vitro reveals common secretion profiles and potential utility as salivary biomarkers. *Oncotarget* **8**, 82459–82474 (2017).

27. Li, J. *et al.* MiR-21 Indicates Poor Prognosis in Tongue Squamous Cell Carcinomas as an Apoptosis Inhibitor. *Clin. Cancer Res.* **15**, 3998–4008 (2009).

28. Li, Y. *et al.* Progressive miRNA expression profiles in cervical carcinogenesis and identification of HPV-related target genes for miR-29. *J. Pathol.* **224**, 484–495 (2011).

29. Lindenbergh-van der Plas, M. *et al.* Identification of Lethal microRNAs Specific for Head and Neck Cancer. *Clin. Cancer Res.* **19**, 5647–5657 (2013).

30. Liu, C.-J. *et al.* miR-31 Ablates Expression of the HIF Regulatory Factor FIH to Activate the HIF Pathway in Head and Neck Carcinoma. *Cancer Res.* **70**, 1635–1644 (2010).

31. Lui, W.-O., Pourmand, N., Patterson, B. K. & Fire, A. Patterns of known and novel small RNAs in human cervical cancer. *Cancer Res.* **67**, 6031–6043 (2007).

32. Manasa, V. G. & Kannan, S. Impact of microRNA dynamics on cancer hallmarks: An oral cancer scenario. *Tumor Biol.* **39**, 1010428317695920 (2017).

33. Manikandan, M. *et al.* Oral squamous cell carcinoma: microRNA expression profiling and integrative analyses for elucidation of tumourigenesis mechanism. *Mol. Cancer* **15**, 28 (2016).

34. Martinez, I. *et al.* Human papillomavirus type 16 reduces the expression of microRNA-218 in cervical carcinoma cells. *Oncogene* **27**, 2575–2582 (2008).

35. Martinez, B. V. *et al.* Circulating small non coding RNA signature in head and neck squamous cell carcinoma. *Oncotarget* **6**, 19246–19263 (2015).

36. Miller, D. L. *et al.* Identification of a Human Papillomavirus–Associated Oncogenic miRNA Panel in Human Oropharyngeal Squamous Cell Carcinoma Validated by Bioinformatics Analysis of The Cancer Genome Atlas. *Am. J. Pathol.* **185**, 679–692 (2015).

37. Nair, J. *et al.* Gene and miRNA expression changes in squamous cell carcinoma of larynx and hypopharynx. *Genes Cancer* **6**, 328–340 (2015).

38. Nohata, N. *et al.* Tumour suppressive microRNA-874 regulates novel cancer networks in maxillary sinus squamous cell carcinoma. *Br. J. Cancer* **105**, 833–841 (2011).

39. Nunez Lopez, Y. O., Victoria, B., Golusinski, P., Golusinski, W. & Masternak, M. M. Characteristic miRNA expression signature and random forest survival analysis identify potential cancer-driving miRNAs in a broad range of head and neck squamous cell carcinoma subtypes. *Rep. Pract. Oncol. Radiother.* **23**, 6–20 (2018).

40. Quabius, E. S. *et al.* miRNA-expression in tonsillar squamous cell carcinomas in relation to HPV infection and expression of the antileukoproteinase SLPI. *Papillomavirus Res.* **4**, 26–34 (2017).

41. Ramdas, L. *et al.* miRNA expression profiles in head and neck squamous cell carcinoma and adjacent normal tissue. *Head Neck* **31**, 642–654 (2009).

42. Rao, Q., Zhou, H., Peng, Y., Li, J. & Lin, Z. Aberrant microRNA expression in human cervical carcinomas. *Med. Oncol.* **29**, 1242–1248 (2012).

43. Salazar, C., Calvopiña, D. & Punyadeera, C. miRNAs in human papilloma virus associated oral and oropharyngeal squamous cell carcinomas. *Expert Rev. Mol. Diagn.* **14**, 1033–1040 (2014).

44. Sannigrahi, M. K. *et al.* DNA methylation regulated microRNAs in HPV-16-induced head and neck squamous cell carcinoma (HNSCC). *Mol. Cell. Biochem.* 1–13 (2018). doi:10.1007/s11010-018-3336-6

45. Scapoli, L. *et al.* MicroRNA expression profiling of oral carcinoma identifies new markers of tumor progression. *Int. J. Immunopathol. Pharmacol.* **23**, 1229–1234 (2010).

46. Schneider, A. *et al.* Tissue and serum microRNA profile of oral squamous cell carcinoma patients. *Sci. Rep.* **8**, 675 (2018).

47. Severino, P. *et al.* MicroRNA expression profile in head and neck cancer: HOX-cluster embedded microRNA-196a and microRNA-10b dysregulation implicated in cell proliferation. *BMC Cancer* **13**, 533 (2013).

48. Severino, P. *et al.* High-throughput sequencing of small RNA transcriptomes reveals critical biological features targeted by microRNAs in cell models used for squamous cell cancer research. *BMC Genomics* **14**, 735 (2013).

49. Vojtechova, Z. *et al.* Comparison of the miRNA profiles in HPV-positive and HPV-negative tonsillar tumors and a model system of human keratinocyte clones. *BMC Cancer* **16**, 382 (2016).

50. Wald, A. I., Hoskins, E. E., Wells, S. I., Ferris, R. L. & Khan, S. A. Human papillomavirus alters microRNA profiles in squamous cell carcinoma of the head and neck (SCCHN) cell lines. *Head Neck* **33**, 504–512 (2011).

51. Wan, Y. *et al.* Salivary miRNA panel to detect HPV-positive and HPV-negative head and neck cancer patients. *Oncotarget* **5**, (2017).

52. Wang, X. *et al.* Aberrant expression of oncogenic and tumor-suppressive microRNAs in cervical cancer is required for cancer cell growth. *PloS One* **3**, e2557 (2008).

53. Wang, X. *et al.* microRNAs are biomarkers of oncogenic human papillomavirus infections. *Proc. Natl. Acad. Sci.* **111**, 4262–4267 (2014).

54. Wang, F. *et al.* Integrated analysis of microRNA regulatory network in nasopharyngeal carcinoma with deep sequencing. *J. Exp. Clin. Cancer Res. CR* **35**, (2016).

55. Wong, T.-S. *et al.* Mature miR-184 as Potential Oncogenic microRNA of Squamous Cell Carcinoma of Tongue. *Clin. Cancer Res.* **14**, 2588–2592 (2008).

56. Wong, N. *et al.* Prognostic microRNA signatures derived from The Cancer Genome Atlas for head and neck squamous cell carcinomas. *Cancer Med.* **5**, 1619–1628 (2016).

57. Xu, Y.-F. *et al.* Identification of miR-143 as a tumour suppressor in nasopharyngeal carcinoma based on microRNA expression profiling. *Int. J. Biochem. Cell Biol.* **61**, 120–128 (2015).

58. Yan, Y. *et al.* Circulating miRNAs as biomarkers for oral squamous cell carcinoma recurrence in operated patients. *Oncotarget* **8**, 8206–8214 (2017).

59. Zhang, Y., Chen, Y., Yu, J., Liu, G. & Huang, Z. Integrated transcriptome analysis reveals miRNA–mRNA crosstalk in laryngeal squamous cell carcinoma. *Genomics* **104**, 249–256 (2014).

60. Zhang, X. *et al.* Regulation of the tumour suppressor PDCD4 by miR-499 and miR-21 in oropharyngeal cancers. *BMC Cancer* **16**, (2016).

61. Zhou, Y., Kolokythas, A., Schwartz, J. L., Epstein, J. B. & Adami, G. R. microRNA from brush biopsy to characterize oral squamous cell carcinoma epithelium. *Cancer Med.* **6**, 67–78 (2016).

62. Zou, A. E. *et al.* The non-coding landscape of head and neck squamous cell carcinoma. *Oncotarget* **7**, 51211–51222 (2016).
